# Understanding barriers and facilitators of integrating management of Moderate Acute Malnutrition (MAM) into the Ethiopian primary health care system using Consolidated Framework for Implementation Research (CFIR)

**DOI:** 10.1371/journal.pgph.0007127

**Published:** 2026-08-14

**Authors:** Alinoor Mohamed Farah, Samson Gebremedhin Gebreselassie, Yakob Desalegn Nigatu, Bilal Shikur Endris, Aweke Kebede, Kemeria Barsenga, Tafara Ndumiyana, Robert Ackatia-Armah, Hiwot Darsene, Beza Yilma Shiferaw, Seifu Hagos Gebreyesus

**Affiliations:** 1 School of Public Health, Addis Ababa University, Addis Ababa, Ethiopia; 2 School of Public Health, Jigjiga University, Jigjiga, Ethiopia; 3 World Food Programme (WFP), Addis Ababa, Ethiopia; 4 Nutrition Coordination Office, Federal Ministry of Health, Addis Ababa, Ethiopia; Islamic Azad University South Tehran Branch, IRAN, ISLAMIC REPUBLIC OF

## Abstract

Existing studies focus on moderate acute malnutrition (MAM) management, with little attention to integrating MAM services into the health system. This study examines barriers and facilitators to integrating m (MAM) services into primary health care. We conducted a qualitative exploratory study at the policy, primary health care, and community levels in six regions of Ethiopia. We used the Consolidated Framework for Implementation Research (CFIR) to guide the development of the data collection tools and the analysis and presentation of the findings. We purposively selected participants and conducted in-depth interviews with 128 participants at the national, regional, district, and facility levels. Hybrid deductive-inductive analysis analysis was applied and the analysis was conducted using NVivo software version 14. Ratings were used to understand how strongly the constructs facilitated or hindered MAM integration, and patterns across levels were compared using heat maps. Integrating MAM management into the health system was considered useful and feasible, with a reliable and adaptable system in place. However, the absence of a dedicated government budget for supply chains, transport and storage services, and supervision creates heavy reliance on external donor funding, undermining government ownership. At the primary care level, understaffing and extensive paperwork and documentation requirements reduce self-efficacy and contribute to inconsistent implementation. These challenges are further compounded by community-level pressures, including misconceptions that the targeted medical supplement is a general food distribution, which increases demand beyond eligibility criteria. Additionally, access barriers such as distance, mobility constraints, and seasonal conditions, along with household dynamics such as sharing of supplements, affect utilization. Integrating MAM management into routine primary healthcare is perceived as valuable and adaptable, with support from existing policies. However, successful and sustained implementation remains constrained by context-specific health system challenges.

## Introduction

Acute malnutrition or wasting is characterized by a low weight-for-length/height z-score and/or a low mid-upper arm circumference (MUAC). Wasting is often subdivided into severe (SAM) and moderate acute malnutrition (MAM) [1]. Although children with moderate wasting experience a notably lower mortality rate than those suffering from severe wasting or nutritional edema, their significantly larger numbers result in them contributing to approximately 30%–40% of deaths attributed to wasting [2].

Globally, recent prevalence estimates suggest that approximately 31 million children under five years of age are affected by MAM [3]. Approaches to managing MAM vary depending on the context, ranging from counselling about healthy diets and medical care to dietary supplementation with specially formulated foods for all moderately wasted children [4]. Until recently, the World Health Organization (WHO) did not have specific guidelines for managing MAM. However, the Essential Nutrition Actions include some recommendations focused on promoting and supporting breastfeeding, as well as providing nutrition education or counseling. In situations of humanitarian crises, where the nutritional and energy needs of moderately malnourished children cannot be fulfilled with locally available foods, specially designed foods can be distributed. These foods are usually in the form of fortified blended foods or ready-to-use supplementary foods, supplied through outpatient-based supplementary feeding programs [5–7].

Despite some progress in reducing child wasting, Ethiopia continues to face a significant burden of acute malnutrition. From 2000 to 2019, the prevalence of wasting decreased by five percentage points, dropping from 12% to 7% [8–11]. However, 7% of children in Ethiopia are still wasted, with 6% being moderately wasted [11]. Thus, Ethiopia’s progress towards achieving the Development Goal (SDG) to reduce the prevalence of wasting to less than 3% by 2030 is inadequate [12].

Ethiopia has implemented interventions to ensure equal access to nutritional services for all children through health facilities and community-based programs [7]. The Health Extension Program (HEP) has played a vital role in extending service coverage to remote and rural areas. Under this program, the Ministry of Health (MoH) established health posts and deployed two Health Extension Workers (HEWs) to each kebele (village) to provide preventive and basic curative services [13].

Prior to 2019, the management of MAM in Ethiopia was overseen by the National Disaster Risk Management Commission (NDRMC), which distributed monthly food rations based on screening data provided by HEWs. However, in August 2019, the Ministry of Health (MoH) introduced a new National Guideline for the Management of Acute Malnutrition, which integrated MAM management into the Ethiopian health system, known as the Integrated Management of Acute Malnutrition (IMAM). This guideline aligned the MUAC admission criteria with WHO recommendations, combined the management of SAM and MAM, and shifted the responsibility for MAM from the NDRMC to the MoH, promoting a unified and systematic approach to addressing wasting in Ethiopia [14]. This implies that the same infrastructure and human resources are used to manage SAM and MAM cases. These changes may impact the capacity of the available infrastructure and warehouse management practices, as more commodities will need to be stored and managed at the facility [15].

Integrating MAM management into the primary healthcare system could support the earlier identification of MAM cases, improve coverage, and enhance the likelihood of timely and appropriate management and follow‑up. However, this integration may also increase the caseload for frontline health extension workers, potentially compromising the quality of care provided to children with SAM and further straining already limited resources in the health system.

Given the current evidence gaps, particularly because most existing studies focus on the effectiveness of specific MAM management approaches rather than their integration into health systems, it is essential to identify the barriers and facilitators to integrating MAM management into primary health care. This study aims to inform strategies for effectively implementing the revised national guideline recommendations within the Ethiopian health system’s capacity. The findings will contribute to the development of evidence-based approaches that optimize MAM management in Ethiopia and similar contexts. Furthermore, the findings will help address existing gaps related to the 2023 WHO guidelines on wasting and nutritional edema, particularly those concerning context-specific implementation challenges in managing moderate wasting.

## Methods

### Ethics statement

This study was approved by the Institutional Review Board (IRB) of the College of Health Sciences, Addis Ababa University, with approval number AAUMF 01–008. All study procedures were performed in accordance with the ethical principles outlined in the Declaration of Helsinki. All participants were informed about the purpose of the study, assured of confidentiality, and provided written consent before participation. Participation was voluntary, and respondents could withdraw at any time without any consequences.

### Study setting

**Fig 1:**
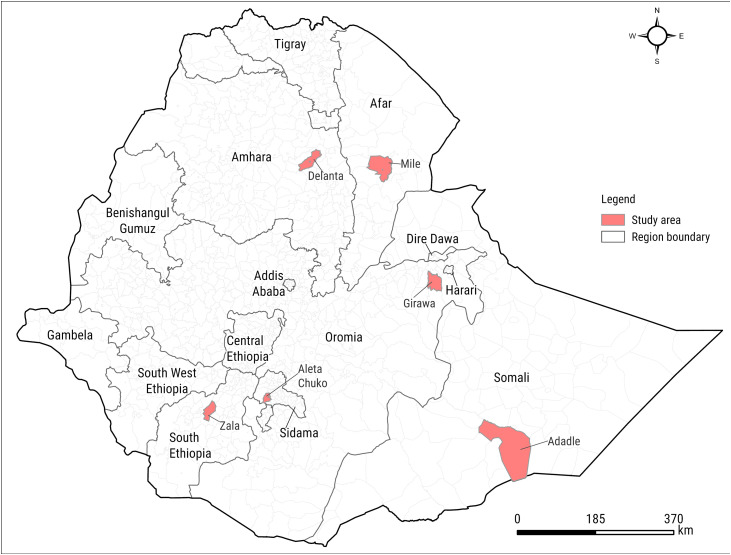
Study areas.

The IMAM program is currently being piloted in 150 districts across six regions: Afar, Amhara, Oromia, Sidama, Southern Ethiopia, and Somali. For this study, six districts implementing the IMAM program–Mile, Delanta, Grawa, Aleta Chuko, Zala, and Adadle–were purposefully selected in consultation with the Ministry of Health (MoH), the World Food Programme (WFP), and UNICEF. These districts were selected based on their context, such as pastoralist, semi-pastoralist, and agrarian. In other words, districts were selected by agro-ecological zone to ensure complete representation, allowing for an understanding of context-specific IMAM implementation (Fig 1).

### Study approach

This study employed a qualitative exploratory approach using semi-structured interviews with key IMAM stakeholders to investigate barriers and facilitators to integrating MAM into the healthcare system at multiple levels (federal, regional, district, and PHCU). This approach was deemed appropriate because there is little or no evidence of barriers and facilitators to integrating MAM into the primary health care system [16]. CFIR framework was used to guide the development of the interview guides, analyze the data, and present key actionable findings. The original version of the CFIR was used. The CFIR was created to systematically assess multilevel implementation contexts to identify factors that could impact the implementation and effectiveness of an intervention [17].

### Participants and recruitment

The study participants were purposively selected from the national, regional, woreda, facility, and community levels to ensure a broad representation of stakeholders involved in the program. A total of 128 in-depth interviews (IDIs) were conducted using a theoretical sampling approach until thematic saturation was reached. Data were collected from February 15, 2023, to April 5, 2023.

### Recruitment of participants

A purposive sampling method was employed to select respondents. At the national level, representatives involved in Ethiopian nutrition policy and practice at the federal level were selected for the study. Key informants (n = 9) were individuals knowledgeable about IMAM implementation, selected from ministries and agencies that had signed memorandums of understanding for IMAM implementation. These include the MoH, the NDRMC, the WFP, and UNICEF. We also interviewed international NGOs, such as Save the Children, Goal Ethiopia, Action Against Hunger, and World Vision. At the sub-national level, in-depth interviews (n = 16) were conducted with regional health bureau (RHB) nutrition team leaders, HEP focal persons, IMAM coordinators, and Child Survival Initiative (CSI/TSF) unit representatives and MCH coordinators. At the district level, interviews (n = 22) were conducted with the Woreda Administration, Woreda Health Office, and Woreda DRM offices. At the PHCU, interviews (n = 58) were conducted with the heads of the health center, HEP and nutrition coordinators, and HEWs at health posts. At the community level, interviews (n = 23)were conducted with Women Development Army (WDAs) members, program beneficiaries including pregnant and lactating mothers and village leaders.

### Data collection: Tools and procedures

The original CFIR framework was used to guide the development of the interview guides. The purpose of these interview guides was to explore potential barriers and facilitators to the integration of MAM services. The development of the data collection tools involved collaborative workshops with key stakeholders engaged in addressing acute malnutrition in Ethiopia.

To ensure contextual relevance, the guides were tailored for different levels of implementation, including the policy (macro), primary health care (meso), and community (micro) levels. At the policy level, participants were contacted by the principal investigator (PI) because many were members of the project technical advisory committee (TAC). Policy-level participants from INGOs and UN agencies were contacted through the MoH. Official letters were sent to institutions at primary health care units and community levels regarding the study. Participants were selected based on their involvement in the IMAM at the policy and implementation levels in collaboration with the MoH.

Prior to each interview, informed consent was obtained in writing after explaining the study’s aims, procedures, confidentiality protections, and participants’ right to withdraw without penalty. When selected individuals declined, replacements were sought only if a key stakeholder group risked under-representation, with the team consulting institutional contacts to identify the alternatives. Interviews were conducted in person at the participants’ private workplaces or remotely by phone or virtual platforms (Zoom meetings) when in-person meetings were not feasible.

Data were collected by 22 trained research assistants. Interviews were conducted in the local languages of Amharic, Afaan Oromo, and Somali. All interviews were audio-recorded, and a standardized transcription protocol was used to ensure consistency. The interview duration ranged from 33 to 127 min, with a mean duration of 59 min.

### Data analysis

The interviewers transcribed and translated the interviews into English while maintaining contextual accuracy. Transcription was completed soon after the interviews to minimize recall bias. All in-depth interviews were transcribed verbatim in the original language and subsequently translated into English by transcribers proficient in both English and the respective local languages (Amharic, Afaan Oromo, and Af‑Somali). Our analysis was based on audio recordings, detailed interview notes, and debriefing field notes. The data were primarily coded using the predetermined codes from the CFIR framework. Three individuals conducted the initial coding, deliberated on any agreements or disagreements, and involved a fourth person to mediate and achieve consensus on the coding. The research team then reviewed and discussed the findings.

Hybrid deductive-inductive analysis was applied [18], beginning with a deductive coding structure informed by the CFIR framework and then guided by emerging data to identify key barriers and facilitators to MAM integration. This method was deemed appropriate because it allowed us to systematically apply CFIR’s predefined domains and constructs to the data while remaining responsive to capture unanticipated barriers and facilitators to MAM integration. After familiarizing ourselves with the data through repeated reading of transcripts, notes, and written responses, we developed initial coding nodes and sub-nodes based on the domains and constructs of the CFIR framework. Units of analysis, such as sentences or longer semantic segments, were coded deductively into these predefined categories. Hybrid deductive-inductive analysis was performed using NVivo software version 14.

We adapted the CFIR rating approach proposed by Damschroder and Lowery to assess the direction (facilitator or barrier) and strength of influence of each CFIR construct. [19]. Ratings were assigned after reviewing qualitative evidence and reflected how strongly the constructs facilitated or hindered MAM integration. We compared the patterns across levels using heat maps. Constructs lacking information were marked as missing, those without a clear influence were coded as 0, and those showing an influence on implementation were rated as weak (+1/–1) or strong (+2/–2) facilitators or barriers. CFIR constructs were rated according to their direction (barrier/facilitator) and strength of influence at each implementation level (policy, PHCU, and community), allowing for a comparison of how implementation determinants varied across the health system. Table 1 defines the criteria used for the rating assignments.

**Table 1:** Criteria used to assign ratings to constructs.

Ratings	Criteria
-2	The construct is a negative influence in the organization, an impeding influence in work processes, and/or an impeding influence in implementation efforts. The majority of respondents describe explicit examples of how the key or all aspects (or the absence) of a construct manifests itself negatively.
-1	The construct is a negative influence in the organization, an impeding influence in work processes, and/or an impeding influence in implementation efforts. Respondents make general statements about the construct manifesting in a negative way but without concrete examples: (1) the construct is mentioned only in passing or at a high level without examples or evidence of actual, concrete descriptions the construct manifests; (2) there is a mixed effect of different aspects of the construct, but with a general overall negative effect; (3) there is sufficient information to make an indirect inference about the generally negative influence; and/or (4) judged as weakly negative in the absence of the construct.
0	A construct has neutral influence if: (1) it appears to have neutral effect (purely descriptive) or is only mentioned generically without valence; (2) there is no evidence of positive or negative influence; (3) credible or reliable respondents contradict each other; and/or (4) there are positive and negative influences that balance each other out, the construct has some positive influence whereas other influences are negative; overall, the effect is neutral.
+1	The construct is a positive influence in the organization, a facilitating influence in work processes, and/or a facilitating influence in implementation efforts. Respondents make general statements about the construct manifesting in a positive way but without concrete examples: (1) the construct is mentioned only in passing or at a high level without examples or evidence of actual, concrete descriptions the construct manifests; (2) there is a mixed effect of different aspects of the construct, but with a general overall positive effect; and/or (3) there is sufficient information to make an indirect inference about the generally positive influence
+2	The construct is a positive influence in the organization, a facilitating influence in work processes, and/or a facilitating influence in implementation efforts. The majority of respondents describe explicit examples of how the key or all aspects of a construct manifests themselves in a positive manner.
Missing	Respondent(s) were not asked about the presence or influence of the construct or, if they were asked about a construct, their responses did not correspond to the intended construct and was instead coded as another construct. Respondent(s)’ lack of knowledge about a This does not necessarily indicate missing data and may instead indicate the absence of the construct.

### Operationalization of CFIR domains (See Table 2)

**Table 2:** Operational definition of the CFIR domains.

CFIR domains	Operational definition
Intervention	Is MAM management considered effective, practical, and worth integrating into the primary healthcare system?
Outer setting	Does the external environment support MAM management with resources, policies, and community demands?
Inner setting	Is the primary healthcare system structured, resourced, and supportive enough to deliver MAM management consistently?
Individuals	Are health workers skilled, motivated, and confident in managing MAM, and how do they perceive MAM management? How does the community perceive MAM management?
Process	Is there a structured and well-managed process for rolling out and monitoring MAM management?

#### Trustworthiness.

This qualitative study was guided by an interpretivist paradigm, and consistent with it, rigor was established using trustworthiness criteria [20]. Specifically, credibility, transferability, dependability, and confirmability were applied to ensure the quality and trustworthiness of the findings. To ensure credibility, the lead authors participated in and supervised the data collection, observed the context during the collection, and provided guidance. The research team engaged in peer debriefing, and interpretations and conclusions were derived from the raw data using direct quotes to elaborate on the codes and categories. To ensure transferability, the team selected representative, information-rich samples from various levels, including those involved in Ethiopian nutrition policy and practice, individuals knowledgeable about IMAM implementation at the sub-national level and within primary health care units, and program beneficiaries. The team offered detailed descriptions to enable the assessment of transferability to contexts. To ensure dependability and confirmability, the principal investigator and two co-investigators conducted a codebook review and established categories, with agreement among the three authors serving as a check for dependability. For confirmability, the categories were presented preliminarily to the research team to validate the analysis process and findings against the quotes from the text. Additionally, another qualitative researcher recoded some meaning units into categories, achieving a coding-recoding evaluation agreement.

## Findings

### Intervention characteristics

a. *Relative Advantage*

Across respondents at the policy, facility, and community levels, the integration of MAM services into the health system was widely viewed as bringing notable advantages over the previous Targeted Supplementary Feeding Program (TSFP) modality. Health workers consistently described the integrated approach as enabling more comprehensive and continuous care for mothers and children. Several participants emphasized that being able to provide both treatment and counseling in one setting improved the quality of care and strengthened relationships with caregivers. For many frontline workers, seeing undernourished children recover within this integrated model was personally rewarding and motivating.

Respondents also noted the practical benefits. Integration has reduced the need for repeated community mobilization, as caregivers now voluntarily participate in the services. Health posts use IMAM contact points to deliver other essential services, such as antenatal care and immunization, improving overall service uptake. In addition, participants reported that the current IMAM approach is more targeted, transparent, and better managed than the former TSFP system, with supplies reaching those who meet the clear admission criteria.

These perceived advantages are reflected in statements such as,

“*The program brings real change to affected children… we consult with mothers every week, and they are happy to collaborate closely with us*.” - Health extension worker

“*Compared to the previous TSF, this is much better because now it is given only to the intended people… mothers and children are measured and only those who fulfil the criteria receive support*.” - Pregnant/breastfeeding woman

Overall, stakeholders described integrated MAM services as improving program quality, strengthening linkages with caregivers, enhancing targeting, and creating opportunities to bundle multiple health services, benefits that were repeatedly contrasted with the limitations of the previous stand-alone TSFP approach.

b. *Complexity*

The implementation of the MAM service was often described as demanding, largely because several components of the work expanded in scope. Health workers repeatedly noted that the amount of documentation required had substantially increased. They must now manage multiple registers, appointment cards, and follow-up cards, sometimes while attending to a large number of caregivers simultaneously. For many, paperwork was described as the most difficult part of their day, especially when working alone.

One health extension worker explained that the number of forms creates considerable pressure, particularly when the caseloads rise.

“*The thing that troubles me now is the recording. There is a lot of it. There is also a record with an appointment card. Again, there is another card. While working on these, when there are too many people, I become stressed, since I am working alone, because I don’t have anyone to help… It bothers me a lot*…” - Health extension worker

Another challenge relates to the involvement of multiple actors in the program. Because the distribution of specialized food items attracts the attention of local leaders, woreda officials, and other stakeholders, health workers must coordinate activities, manage expectations, and respond to various demands. This adds layers of administrative work that are not directly related to clinical service delivery. As one respondent described, the program required more than simply providing care:

“*To successfully implement and increase the coverage of this program, several tasks must be accomplished. First, the screening process should be conducted appropriately, and reporting should be done on time according to a specific schedule. Secondly, there needs to be coordination with various stakeholders, and specific responsibilities should be assigned to health centers, kebele leaders, and woreda officials*.” - Health extension worker

c. *Evidence strength and quality*

Stakeholders consistently expressed strong confidence in the benefits of integrating MAM services into the health system, describing the program as effective, reliable, and grounded in interventions that they believed produced meaningful improvements for malnourished children and pregnant and lactating women. Respondents highlighted that children recover more quickly when they receive products such as RUSF and CSB++, and many noted that early identification and management through an integrated approach helps prevent deterioration into severe malnutrition. Several participants also emphasized that offering services in one location improves continuity of care, strengthens maternal and child health services, and supports better follow-up.

This confidence was supported by observations of reduced severe acute malnutrition admissions and perceived improvements in child health. Some respondents also described how the integrated program strengthened the health system by facilitating access to other essential services, improving screening coverage, and enabling health extension workers to engage more efficiently with caregivers, who now come forward more readily for nutrition and related services. As one health center head explained,

“*The decrease in SAM rate is one of its benefits… other things that come in relation with malnutrition like diarrhea will also decrease*,” - Health center head

A regional technical advisor similarly noted that

“*IMAM had a very good impact on maternal and child health integration… mothers come easily; it has increased the community participation*.”- Regional technical advisor

d. *Adaptability*

Several respondents described how MAM management was adjusted to fit day-to-day service delivery needs. A commonly mentioned adaptation was the use of community volunteers to support health extension workers with screening, referral, and mobilization. This approach was seen as an important strategy for easing workload pressures and helping prevent burnout, especially in areas with high caseloads or where HEWs work alone. The task-sharing arrangement allowed HEWs to focus more on treatment and reporting while maintaining service.

“*To avoid or manage HEW staff burnout we select three CVs from each sub‑kebele that help and work with HEWs; that is a way we tried to manage staff burnouts*.”- Kebele leader

Respondents also noted that despite the multiple steps involved in diagnosing and managing moderate acute malnutrition, the service aligned relatively well with existing structures. Many felt that MAM management fits naturally alongside SAM management and can be incorporated without major disruption to the established workflows. This perceived compatibility made it easier for staff and partners to adopt the integrated model and helped reinforce the sense that the approach was workable within routine health system functions.

“*I feel MAM can easily fit into the health system and it has fitted easily based on what we see in the field; it is just as good as what is happening with SAM*.”- Implementing partner

e. *Cost*

Funding limitations have been widely described as a major obstacle to sustaining MAM management in the primary healthcare system. Local governments rely almost entirely on partners to cover operational costs, such as supervision, transportation of nutrition supplies, and routine coordination, which makes it difficult to run the program in areas where partner support is lacking.

“*There is no dedicated budget planned to support for IMAM implementation from the government*.”- Woreda Disaster Risk Management focal

Similar gaps have been reported at the national level, where the Ministry of Health covers salaries but does not allocate funding for the commodities, operational expenses, or logistics required for MAM. Most financing still comes from external partners, making the program dependent on vertical funding streams. Simultaneously, emerging signs of domestic investment are evident, including the recent decision to allocate a matching fund for RUTF procurement, which is viewed as a modest yet significant step toward government ownership.

“*With the exception of human resource there were no other contribution from MoH or the government in general*.”- Ministry of Health Nutrition Coordinator

“*We… allocated a matching fund… around 1.3 million USD… it may not be enough, but it is a good start and [we are] planning to increase the matching fund in the future*.”- Ministry of Health Nutrition Coordinator

### Outer setting

a. *Patients’ needs and resources*

Respondents described several factors that made it difficult for caregivers, particularly those in remote or pastoralist areas, to access and use MAM services. Long walking distances to health posts, crowded service points, and lengthy waiting times were consistently mentioned as barriers that discouraged timely attendance, especially when caregivers had to visit twice a month. Interruptions linked to other health campaigns or supply shortages further complicate access, leaving some beneficiaries unable to receive supplements as expected.

“*We did not receive the supply for the next month because the HEWs are engaged in an ongoing campaign. It would be nice if it wasn’t discontinued because of the campaign*.”-Caregiver, FGD

Physical access challenges were particularly pronounced in pastoralist communities where mobility patterns limited service coverage. Several respondents noted that a significant proportion of eligible households move frequently with their livestock and thus miss screening sessions or scheduled distribution days, reducing the program’s reach.

“*Most people are mobile due to their lifestyle and miss this service*.”- Woreda administrator

Caregivers also raised concerns about household-level barriers, including the difficulty in preventing the sharing of supplements among children, particularly twins or siblings of different ages. Additionally, suggestions were made to extend the duration of support for the lactating mothers.

“*It would be good if lactating mothers stay nine months in the program rather than four*.”-Health center head

b. *External policy and incentives*

Respondents consistently noted that there were no formal incentive mechanisms linked to the implementation of IMAM beyond per‑diem payments for attending training or review meetings organized by the government or partners. Health workers generally do not expect direct incentives, and some officials explicitly discourage such expectations, emphasizing that staff participate in multiple programs where small monetary benefits may be occasionally provided.

“*The head of the sector tells us to do our job and not seek any incentives… If you don’t receive incentives from one program, you will get from other programs*.”- Woreda Disaster Risk Management focal

Several external and environmental conditions shape the effectiveness of program implementation. Poor road infrastructure, long travel distances, and seasonal rains frequently disrupt last-mile distribution, particularly in remote pastoralist areas. These challenges not only affect the transport of nutritional supplies but also impede routine supervision and follow-up by Woreda teams and partners.

“*Yesterday I went for supervision, and I could not reach my destination because of rains… the top challenge is road and distance*.”-Woreda administrator

Policy actors described the national environment as broadly supportive of nutrition priorities, with recent increases in domestic funding, such as matching funds for RUTF procurement, representing a positive shift.

“*The budget allocated is not enough, but we have seen some improvement on the amount allocated over three years especially for the Seqota since the government has allocated 10 to 12 million USD for the last three years*.”- Ministry of Health Nutrition Coordinator.”

### Inner setting

a. implementation climate
  i. *Relative priority*

Respondents generally considered the management of moderate acute malnutrition an important service within the health system because of its direct impact on child survival. However, they also explained that the degree of attention it receives often depends on external factors such as partner support, supply availability, and competing program demands. In many instances, routine services, especially vaccination and campaign‑based activities, take precedence, leaving MAM implementation adjusted around these higher‑priority tasks.

“*To be honest, vaccination is given priority. However, we will also prioritize the IMAM program*.”-Health extension worker

Despite these competing pressures, some facilities have consistently maintained attention to MAM activities through structured scheduling. In such cases, the managers ensured that at least one health extension worker remained available to provide services when others attended training or meetings. This type of support helped ensure that MAM services were delivered as planned and not postponed because of conflicting obligations or other issues.

“*They give priority to MAM… one of us remains in the office and we provide MAM service when other HEWs leave for training and review meetings… We don’t schedule anything on the MAM day*.”-Health extension worker

ii. *Organizational incentives and rewards*

Respondents noted that no formal incentive systems were linked to performance in MAM service delivery. Health workers do not receive rewards, promotions, or salary adjustments for their contributions to the IMAM program. Instead, any financial benefits they might receive are indirect, stemming from participation in training or review meetings organized by the government or partner organizations. In some settings, certificates of appreciation or public recognition are used to motivate high-performing staff, and these gestures are considered meaningful, even in the absence of financial rewards.

“*A person needs recognition more than financial incentive… it’s really motivating if someone is given a trophy or a certificate as a good performer*.”- Regional Health Bureau HEP Coordinator

When asked what would improve motivation, the respondents consistently highlighted the importance of supportive supervision, manageable workload, and opportunities for career advancement. Many HEWs expressed a desire for transfers, additional staffing to reduce the burden of working alone, and improved wages to match their responsibilities. Others have noted that periodic access to education or training opportunities serves as a strong motivator and helps to retain staff.

“*What can motivate us… can be the trainings, wage increment*.”- Health extension worker

iii. *Goals & feedback*

Respondents described some efforts to monitor MAM activities and review performance indicators at the woreda level; however, many felt that the follow-up mechanisms were insufficiently consistent or effective. Although reports are shared with relevant offices and periodic supervision is conducted, health extension workers have explained that these visits do not always translate into practical support or actionable feedback.

“*After we finalize the monitoring and supervision, we present it to all the concerned stakeholders… We use performance indicators*.”- Woreda nutrition focal

On the frontline, health extension workers expressed concern that supervision could be brief or irregular, offering little opportunity to discuss challenges or align efforts with the program goals. This inconsistency affects the extent to which goals are communicated and how feedback is used to improve the service delivery.

“*They come sometime and simply go back*.”- Health extension worker

iv. *Learning climate*

Respondents described a generally supportive learning environment, highlighting that PHCUs, woredas, and regional teams conducted periodic visits to guide the health extension workers and assess MAM performance. These visits were considered beneficial for reinforcing expectations regarding screening, defaulter tracing, and commodity management, as well as for identifying gaps that need to be addressed. In several areas, supervisors utilized routine data and key indicators to structure their discussions with staff, which helped make feedback more concrete and actionable.

Several respondents highlighted the value of mentorship provided alongside supervision, explaining that it strengthened health workers’ confidence and ability to manage cases correctly. Monthly mentorship sessions were mentioned as particularly useful because they allowed for more in-depth support than brief supervisory visits.

“*The IMAM indicators are used during supportive supervision… This monitoring and supportive supervision is followed by mentorship which is conducted once each month*.”- PHCU‑level HEP supervisor

b. Readiness for implementation
  i. *Available resources*

Respondents generally welcomed the integration of MAM services into routine health activities; however, many emphasized that the actual readiness to implement the program was constrained by limited staffing, high workload, and tasks that extended beyond traditional responsibilities. Health extension workers and facility staff described the distribution of nutrition commodities as a unique responsibility that added administrative and logistical demands to an already full workload. In several areas, staff shortages meant that health extension workers were working alone, making it difficult to manage screening, counselling, documentation, and distribution effectively.

“*Some health extension workers view the project as an additional task rather than as part of their responsibilities. Additionally, there are supply shortages and delays in delivery*.”- Woreda health office head

Beyond human resources, respondents highlighted several operational challenges that limited preparedness at the facility and Woreda levels. These include insufficient storage space for nutrition supplies, lack of funds to build or renovate warehouses, transport constraints, gaps in training, and limited access to safe water at health posts. Regional officials noted that some of these needs exceeded local capacity and required national support. Supply interruptions were cited across all regions, with delays, stockouts, and near-expired products disrupting service provision, issues linked to broader funding shocks, transportation problems, and accountability gaps within supply chains.

“*In general, it requires national readiness. The burden on the health extension workers and the storage space need attention from the national level. Sometimes it’s beyond the region’s capacity*.”- Regional health bureau nutrition focal

Despite these constraints, respondents also identified areas where emergency nutrition responses and partner engagement had strengthened local systems. The integration of MAM services within existing reporting, supervision, and review structures was considered a positive development. At the same time, funding shortages continued to affect essential operations, such as storage maintenance, offloading, and the guarding of supplies. Transportation challenges and logistical bottlenecks, exacerbated by rising fuel prices, poor road conditions, and global supply disruptions, were consistently mentioned as barriers undermining the timely distribution of commodities.

*There was significant improvement in funding, and the issues of Ukraine started to shock us and there was no commodity*.” – IMAM implementor

ii. *Leadership engagement*

Respondents described leadership engagement as mixed, with strong involvement from partners and varying levels of commitment from the government structures. Leaders played visible roles in managing the transport of supplies, enforcing service continuity, and participating in coordination platforms such as the IMAM technical working group. These efforts helped maintain the program’s momentum despite operational challenges.

“*The government takes the leadership role in the transportation of the supplies up to the health posts… The government takes the responsibility if there is interruption of the service*.”- Regional health bureau IMAM technical advisor

Respondents noted that leadership engagement had not yet translated into dedicated budgeting for the MAM program. Key operational needs, such as supply procurement, storage construction, offloading, transport from Woredas to Kebeles, and capacity-building activities, remained largely dependent on partners. Although national coordination mechanisms are strong and the Ministry of Health plays an active convening role, financial contributions to the program remain limited, creating gaps that hinder full ownership and sustainability.

“Transportation and review meetings are covered through partner organizations.”- Health Center Head

“*The Ministry of Health is driving a lot of work… facilitating and organizing the technical working group for IMAM. Almost all the time there is agenda on IMAM*.”- Implementing partner

iii. *Access to knowledge and information*

Respondents described health extension workers as generally knowledgeable and capable of delivering integrated MAM services; however, they also emphasized notable variations in technical skills, particularly among newly deployed staff. Many health extension workers had received only an initial round of training when the program began, and some newer workers had joined without formal instruction on the updated IMAM guidelines. Orientation was often used as a substitute for full training, which limited confidence in areas such as diagnosis, counselling, and correctly completing program forms.

“*Training was provided only once. There are also new health extension workers that didn’t take training, but we gave them orientation and they started their job*.”- Health center head

Gaps were also noted in the use of the district health information system (DHIS-II) and in recording and reporting practices. Several respondents explained that many health extension workers had not received formal training on the DHIS-II platform and were learning informally while already carrying out service delivery. This has contributed to inconsistencies in documentation and challenges in maintaining accurate records, tasks that are central to managing MAM caseloads and monitoring program performance.

“*Most of them did not receive training on DHIS‑II. Some of them are new and they didn’t take the training*.”- Health center head

“*The problem is reporting and recording… at the delivery point there is a problem with recording by some of the workers*.”- Health extension program supervisor

c. Network and communication

A range of communication channels that link health extension workers, communities, and administrative structures in support of MAM implementation was identified. Regular community meetings, Kebele gatherings, and interactions between health extension workers and local actors provide opportunities to share updates, discuss challenges, and reinforce expectations around service delivery. In addition to these in‑person platforms, several areas use digital communication, particularly WhatsApp groups, to share evidence of activities, coordinate distributions, and exchange rapid updates between health posts, health centers, woreda offices, and regional teams.

“*We have a WhatsApp group; we use it when we go out to send pictures and videos as evidence and for reporting*.”- Health extension worker

These communication mechanisms also strengthened local accountability and the program’s visibility. Participants described how issues related to service delivery, community health, and MAM performance were raised during kebele meetings or sector discussions, ensuring that health extension activities were connected to the wider community structure. Caregivers also noted that community improvement forums created space for engaging with health workers and understanding ongoing priorities.

“*We have meetings with the administration… the administration always mentions our job in that meeting*.”- Health extension worker

### Characteristics of Individuals

a. *Knowledge and beliefs about the intervention*

Health extension workers generally expressed a positive attitude toward the MAM program, highlighting their strong appreciation of its role in enhancing child nutrition and the high value of the specialized foods it provides. However, they also pointed out that misunderstandings within the community pose ongoing challenges. Many caregivers initially believed that the program was meant for all mothers and children, assuming that it operated as a blanket food distribution rather than a targeted therapeutic service. This misconception led to confusion among Kebele leaders and continuous pressure on HEWs to admit beneficiaries who did not meet the criteria.

“*Previously there was misunderstanding… they assumed that IMAM supplies are under their control, but they are informed not to interfere*.”- Health extension worker

Local authorities and caregivers frequently referred to the program as “distribution,” which reinforces the expectation that everyone should receive food, regardless of nutritional status. This perception sometimes discourages caregivers from using other health services if they or their child are not eligible for MAM services, creating tension between service providers and the community. HEWs reported that some families become upset when children do not pass screening or when supplies cannot be provided.

“*Most people do not think this supplement is a therapeutic food; they perceive it as a donation. If their child does not pass the screening, they get mad at the health extension worker*.”- Health extension program supervisor

These misconceptions also contribute to community pressure on HEWs to enroll non-eligible beneficiaries, making adherence to the program criteria difficult in some kebeles. Although ongoing community sensitization efforts have begun to reduce misunderstandings, respondents stressed the need for a more comprehensive orientation to clarify program objectives, reinforce that the supplements are therapeutic, and align expectations with the intended purpose of MAM services.

“*Everyone wants to be enrolled into the program*.”- Women Development Army

b. *Self-efficacy*

Respondents described notable variations in the confidence and ability of health extension workers to carry out different aspects of the MAM program. Health extension workers were generally viewed as effective in distributing supplies and managing beneficiaries during routine sessions but were less consistent in meeting screening targets and completing documentation accurately. Supervisors noted that screening performance often improves during campaign periods but declines during routine months, reflecting uneven motivation and a limited capacity for sustained outreach. Gaps in data recording and reporting were also highlighted as areas where some health extension workers required additional support.

“*The problem is that they don’t do target screening and they always need our help… the screening performance increases during the campaign, and it drops if there is no campaign*.”- Health extension program supervisor

Despite these challenges, many health extension workers expressed strong commitment to the program and increasing confidence in delivering MAM services as they gained experience. Some initially perceived the work as an added burden; however, over time, they came to value the program’s impact and felt motivated to continue it. However, the overall workload, including frequent distributions, counselling, and documentation, was widely viewed as heavy, creating pressure that affected performance. Supervisors and facility heads attempted to ease these demands by deploying support staff during busy periods; however, high caseloads and competing priorities remained persistent.

“*Previously we believed that this program is extra load… but now we don’t feel any load, and we have commitment to perform this program*.”- Health extension worker

It was also emphasized that program demands often compete with other essential tasks, such as vaccinations and routine maternal and child health services. This competition for time sometimes forces health extension workers to rush screening or compromise the documentation quality. Although the technical ability to implement MAM services is present, the combination of workload, staffing gaps, and competing priorities influences how consistently health extension workers feel able to perform at the expected standards.

“*There are times when screening must be rushed due to other services that must be provided on the same day, such as vaccination*.”- Health extension program supervisor

### The implementation process

a. *Planning and reporting*

Recurring challenges in aligning reported caseloads, monthly supply allocations, and the actual number of beneficiaries seeking services have been identified. Supplies delivered to health posts are based on the previous month’s caseload, which often underestimates current needs and results in shortages when new admissions unexpectedly increase. This mismatch frustrates both providers and caregivers, leading to service interruptions and complaints at distribution points.

“*The supply sent to us for January is based on the report we submitted in December… If we report 200 children in December, then more than 200 may be found in January*.”- Health extension worker

MAM indicators have not yet been integrated into DHIS‑II, and many health extension workers have not received formal training on digital reporting tools, resulting in incomplete or inconsistently filled community cards and registration records. These gaps undermine the reliability of routine data and slow progress toward a unified information system.

“*MAM data has not yet been integrated into DHIS‑II… We have had a lot of advocacies and discussions with the Ministry of Health*.”- IMAM implementor

b. *Engaging*

Respondents described varying levels of engagement among actors involved in delivering the MAM program. Health extension workers receive support from women’s development army groups, village leaders, kebele administrations, and district-level sector offices, particularly in community mobilization, organizing gatherings, and assistance during distribution days. These actors help increase participation and facilitate communication between the health system and the community. However, respondents noted that coordination across sectors at the woreda level, especially between the health and agriculture offices, remains inconsistent, limiting the overall effectiveness of these engagements.

“*The coordination of multi sectors is strong at the higher level but becomes loose as you go downwards… further attention should be given for effective implementation of the program*.”- Regional health bureau nutrition focal

Although community acceptance of the program is high, the level of direct involvement of caregivers, pregnant, and breastfeeding women in planning or decision-making remains minimal. Local leaders and volunteers play important supportive roles by assisting health extension workers in spreading messages, organizing community meetings, and helping manage crowds during distribution sessions. These contributions strengthen community linkages, even in the absence of formal participation structures.

“*Village leaders are also helping us now*.”- Health extension worker

c. *Executing*

Respondents reported that the day-to-day implementation of the MAM program did not always align with the standard protocol for admission, counselling, follow-up, and monitoring. While beneficiaries generally receive their rations quickly during distribution days, the process often prioritizes speed over thorough assessment or counselling, largely due to high caseloads, limited staff, and the pressure to move long queues efficiently. Health extension workers described situations in which screening, advising, and follow-up checks were rushed or inconsistently performed, and where local authorities or community expectations added further pressure to distribute supplies rapidly rather than following the prescribed steps.

“*They give the products to us immediately… they give for the ones who come first depending on who comes first*.”- Caregiver

Adherence to the protocol was affected by logistical and structural challenges. The poor storage capacity of health posts meant that when contractors delivered commodities without notice, and sometimes in large quantities, health extension workers were forced to distribute all supplies simultaneously to avoid spoilage or theft of the supplies.

“*Every day the service for children is interrupted… sometimes we miss two to three months… we keep on waiting until it comes again.”-* Pregnant/breastfeeding women

In combination, workload pressures, inadequate infrastructure, and coordination gaps contributed to a delivery approach that was functional but not fully aligned with the standards expected for screening, follow-up, and counselling. While the system succeeds in distributing supplies, respondents emphasized that without addressing these operational bottlenecks, achieving consistent, protocol-driven implementation will remain challenging.

d. *Reflecting and evaluating*

Participants across community and service delivery levels reflected on the progress and quality of integrated MAM services, generally recognizing the program’s value in supporting the recovery of malnourished mothers and children. Many caregivers expressed appreciation for receiving timely and appropriate supplements, describing the service as meeting their immediate household needs.

“*This service meets our needs because we are given the plumpy sup that we need*.” Pregnant/breastfeeding woman

In contrast, concerns regarding program ownership and accountability emerged. A few woreda administrators perceived IMAM as primarily owned or driven by external partners, which may weaken the long‑term commitment and hinder efforts to embed the program fully within routine government systems.

### A multi-level CFIR rating

a. Macro level (policy)

**Fig 2:**
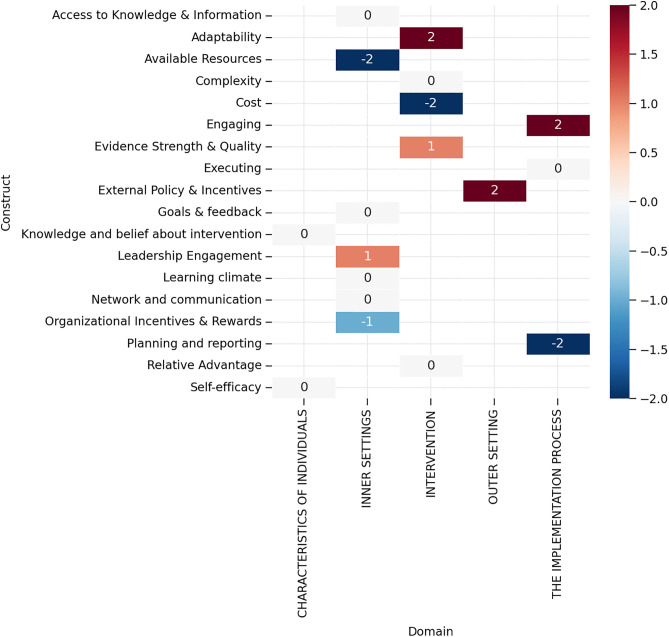
CFIR rating - Macro (policy) level.

External policy and incentives remain a major enabling factor, and leadership engagement is positive, signaling a clear commitment to MAM integration at the national and subnational levels. However, cost and available resources are strong barriers, reflecting limited government funding and an over-reliance on partner support. Planning and reporting are alsostrong barriers because MAM indicators are not integrated into DHIS‑II, forcing parallel reporting systems and undermining coherence between planning and routine performance monitoring (Fig 2).

b. Meso level (primary health care units)

**Fig 3:**
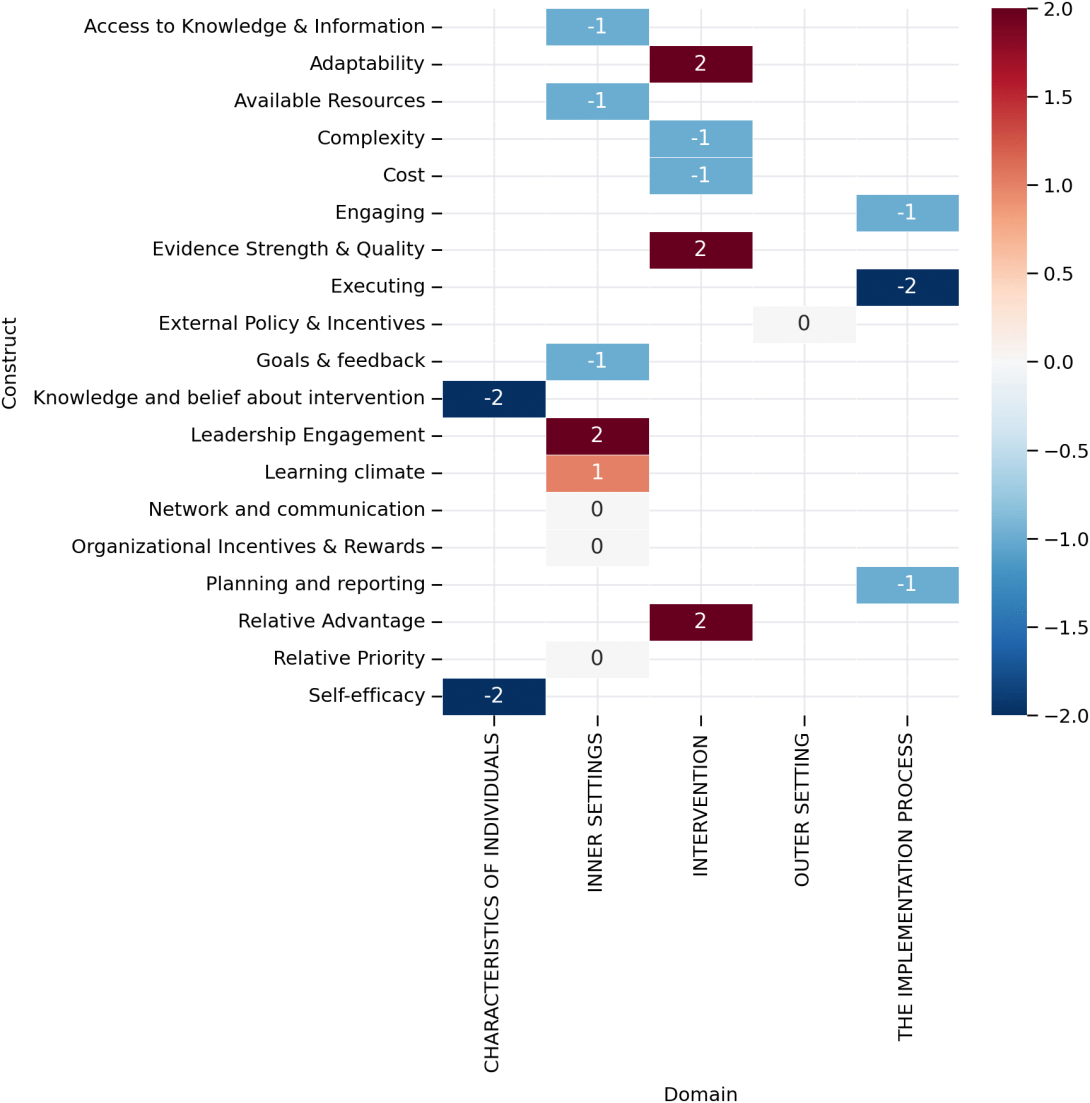
CFIR rating - Meso (primary health care units) level.

Facility-level factors remain the primary implementation bottleneck, with several constructs acting as barriers to fidelity. Executing is a strong barrier, showing rushed in MAM management processes, limited counselling and follow‑up, and disruptions from storage shortages and supply chain breakages. These pressures are reinforced by self-efficacy, goals and feedback, access to knowledge and information, and available resources, illustrating gaps in staffing, skills, supervision, and operational readiness at the primary care level. Despite these constraints, strong enablers included relative advantage, adaptability, and leadership engagement, reflecting the integration value and supportive local leadership. However, these enablers are outweighed by operational and logistical barriers, resulting in implementation that is functional but unable to meet the protocol standards (Fig 3).

c. Micro (community) level

**Fig 4:**
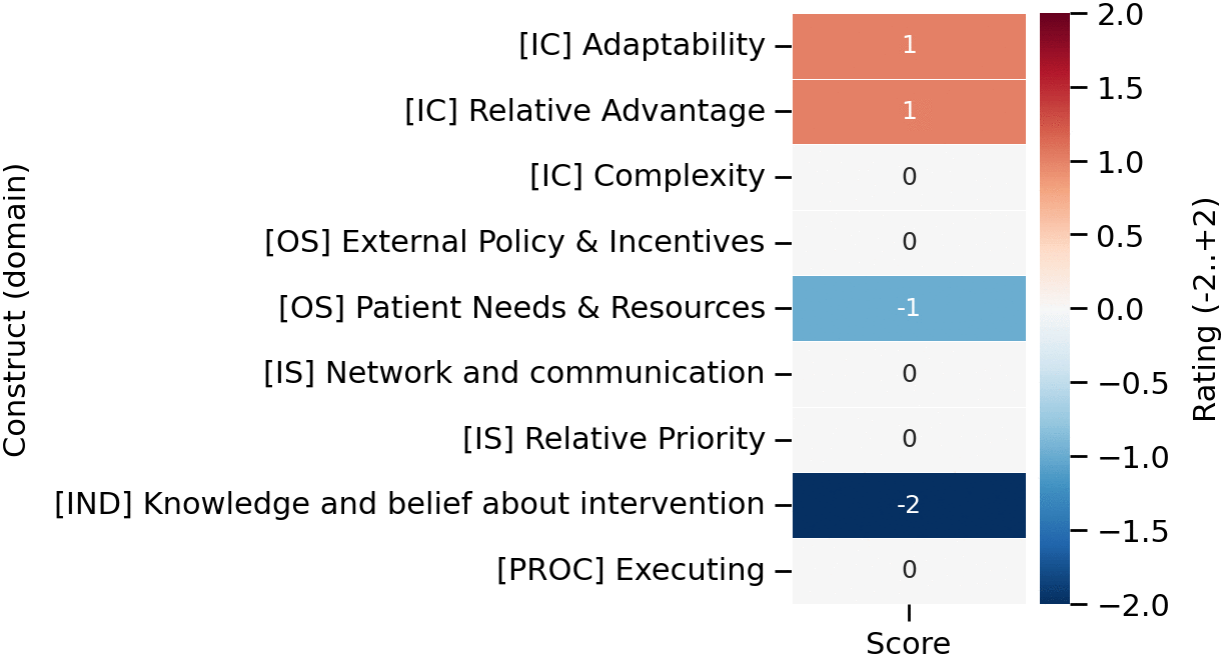
CFIR rating - Micro (community) level.

The community interface is shaped by expectations and accessibility. Knowledge and belief about the intervention were strongly negative, misperceiving supplements as “distribution” rather than suppliment. Modest facilitators exist (relative advantage, adaptability), but knowledge gaps dominate community-level experience and pressure frontline workers (Fig 4).

## Discussion

This study synthesized the barriers and facilitators of integrating the MAM program across the five domains of the CFIR framework. By examining the intervention characteristics, outer and inner settings, characteristics of individuals, and the implementation process, key barriers and facilitators that influence the program’s implementation were identified. The analysis highlights the complex interplay between various elements of the healthcare system and community dynamics, providing valuable insights for improving the management of MAM in Ethiopia.

The IMAM program is favored over standalone TSFP for improved targeting and service accessibility. Access to facilities is a determinant of utilization [21]. A review of healthcare systems showed that establishing a network of facilities increases coverage of underserved populations [22]. Integrating screening and immunization in a single visit reduces missed opportunities and improves efficiency and service delivery. Scaling up nutrition interventions and integrating nutrition-specific interventions into health programs could present opportunities in low- and middle-income countries [23]. However, the increased HEW workload from the MAM program may compete for time and resources, affecting services and causing burnout, reduced effectiveness, and lower job satisfaction.

Health workers face a significant burden from documentation and record-keeping, which is time-consuming and stressful, especially when managed single-handedly. A key limitation is time, as documentation cannot be completed after all clinical activities [24]. Consequently, too much time is spent recording, leaving less time for patient care [33] and [25] and causing incomplete records [26]. Overburdened workers and resource constraints commonly hinder health programs in developing countries [27]. Effective implementation requires workload management, adequate training and resources. Although complex strategies often have a significant impact, simpler approaches can be equally effective; training alone for community health workers has a small effect, but support and training improve outcomes [28].

The adaptability of the MAM program is highlighted by the task sharing between community volunteers and HEWs. This approach improves staff management and increases the efficiency of service delivery, ensuring the program’s sustainability, even with limited human resources. Task shifting can alleviate coverage bottlenecks and promote equity by delegating tasks traditionally handled by qualified health and nutrition professionals to individuals with less formal training. This strategy holds promise for enhancing both the operational efficiency and outreach capacity of the delivery channel [29].

The engagement of both formal and informal actors, such as the Women’s Development Army (WDA), sector offices, local kebele officials, village leaders, and influential individuals, is crucial for effective community mobilization and support. HEWs, working alongside WDA, can significantly improve health service coverage, contributing to the increased uptake of health services [30]. In resource-poor settings, reducing maternal and child mortality depends heavily on community-based interventions that foster cooperation and collaboration among diverse actors [31]. Evidence shows that culturally tailored interventions are more likely to be well received and effective in changing health behaviors [32].

The lack of standardized incentives for health workers, beyond occasional monetary benefits from training and review meetings, can cause low motivation and high turnover, affecting the stability and quality of services. Similar challenges have been reported in low-resource settings, where health workers often lack formal incentive structures [33]. Both financial and non-financial incentives are critical for improving job satisfaction and reducing turnover among health workers. Incentives, including training, job advancement, recognition, mentorship, and supervision, can improve workers’ satisfaction and performance workers [34,35], whereas increased workloads and payment delays can reduce motivation and performance [36,37].

The study findings revealed that, although some monitoring and supervision efforts exist for the MAM program, HEWs are concerned about its effectiveness. Inconsistent supervision may result in missed opportunities to address challenges and improve performance. HEWs also perceive some monitoring visits as superficial, with supervisors visiting briefly without meaningful engagement. Despite its role, supportive supervision is not well understood in the healthcare context [38,39]. Although visits are scheduled, evidence suggests that they often do not occur as planned because of conflicting responsibilities, limited finances and transportation, and accessibility issues [39]. Addressing these shortcomings requires supportive supervision models aligned with factors affecting health workers’ motivation, satisfaction, and performance. The approach in resource-constrained countries is inadequate, requiring improved human resource management, management support, supervision, and feedback to improve retention and quality of care [40].

Insufficient funding for MAM implementation poses a significant barrier, as the government heavily depends on external partners for financial support. This reliance on external funding risks the program’s continuity and effectiveness if such support diminishes or ceases to exist. Limited resources in low-income countries make it challenging to sustain nutrition programs in the long run. Therefore, it is crucial to assess the economic feasibility and long-term impact of such initiatives to ensure their success [27,41].

Interruptions in nutrition commodities can undermine the delivery and accessibility of MAM programs. These disruptions were caused by funding shortages linked to events such as the war in Ukraine and transportation issues, including delayed deliveries due to fuel price fluctuations. Reliable distribution is essential for maintaining program efficacy and ensuring care. Nutrition commodities must be available within functioning health systems at all times, in adequate amounts, in the appropriate dosage, and with assured quality [42].

Poor road conditions, especially during the rainy season, create significant barriers to the last-mile distribution of nutritional commodities and supervision activities necessary for program oversight. These logistical challenges can delay or prevent nutritional commodities from reaching their intended destinations. Without proper infrastructure and logistical support, the efficient operation of these programs is hindered, making it challenging to reach people in need [43].

For the program to be sustainable, the government must strengthen its commitment by allocating budgets for nutrition commodities, infrastructure, and capacity building. Stable and consistent health governance and leadership significantly affect the success of programs [44]. Therefore, improving coordination among stakeholders, ensuring government ownership, and integrating IMAM into broader health strategies are crucial for the program’s long-term success.

The involvement of district-level managers and leaders helped prioritize the MAM program; however, it is often deprioritized in favor of other health initiatives, such as immunization campaigns. This is a common issue in many public health programs, where resources and attention are diverted to more immediate or high-profile diseases [45]. While health campaigns effectively target specific needs, they can divert healthcare workers from their regular duties, causing temporary halts in ongoing health services [45,46]. Thus, campaigns should systematically monitor their impact on routine services and develop concrete strategies to mitigate potential adverse effects [46].

Communities and authorities often misinterpreted the MAM program as food aid rather than a supplementary intervention for malnutrition, likely because HEWs provided inadequate information. Our study echoes the findings from Rwanda, where ambiguous information on the distribution and eligibility criteria for nutritional supplements led beneficiaries to fail to collect or register for them. Despite clear guidelines and training for healthcare staff and community health workers, the challenge remains to ensure that beneficiaries understand the program [47]. Mass media campaigns can help, but their success depends on nutrition commodities and access to services [48,49].

Furthermore, economic constraints have left households unable to adequately provide food for their members, often resulting in the sharing of nutritional supplements intended for malnourished children and mothers. This practice can lead to a slower response to supplementation, necessitating efforts to enhance patient compliance and reduce supplement sharing [50]. Furthermore, this scarcity impacts the proper implementation of programs, such as ensuring the adequate intake of FBF by the target beneficiaries, as these resources are often shared among household members [51].

Drought conditions force pastoralist communities to migrate in search of pasture and water, distancing them from health services and increasing default rates in the MAM program. This disruption highlights the potential of Mobile Health and Nutrition Teams (MHNTs) to provide essential services to remote areas, although they are not widely implemented in developing countries [52,53]. Despite significant investments in combating child wasting, the increased frequency and intensity of environmental and human-made shocks have hindered progress, emphasizing the need for more resilient strategies to address these challenges [54].

Despite the critical role of beneficiaries, including caregivers and PBWs, their engagement in program design, planning, and implementation is lacking. Beneficiary and local stakeholder participation in community-based interventions underscores engagement in community empowerment. This empowerment involves individuals gaining information, skills, and confidence, enabling them to control their life decisions. This process can occur at the individual, organizational, and community levels [55]. This principle reflects the Alma Ata Declaration of Primary Health Care, which emphasizes people’s right and duty to participate in the planning and implementation of their healthcare [56]. This aligns with the goals of self-reliance and health equity [57].

Although integrating MAM management into the health system is perceived as valuable, adaptable, and policy-supported, evidence gaps remain regarding how integration can be implemented and sustained in resource-constrained settings. Future research should move beyond barriers and facilitators to prospectively evaluate which strategies work, for whom, under which conditions, and at what cost, thereby generating actionable evidence to support the effective, scalable, and sustainable integration of moderate wasting management into primary health care.

This study has high credibility for several reasons. First, the study triangulated perspectives from various stakeholders at different levels of the health system across six districts in six Ethiopian regions. This provided a rich and nuanced understanding of the challenges and opportunities for integrating MAM management into primary healthcare systems. Additionally, CFIR was employed as a guiding tool for data collection and analysis, ensuring a systematic exploration of the factors influencing the integration of MAM services.

Our study has several limitations. First, qualitative data were collected retrospectively from stakeholders directly involved in the IMAM implementation. Stakeholders indirectly involved, including representatives from ministries responsible for finance, water, and agriculture, were excluded; thus, important multi-sectoral perspectives may have been missed. Second, the study used the original rather than the updated CFIR, possibly limiting the capture of refined constructs and domains. Nevertheless, it provides a systematic framework for analyzing key barriers and facilitators across health system levels.

## Conclusion

Integrating MAM management into routine primary healthcare is perceived as valuable and adaptable, supported by the policies. However, effective and sustainable implementation remains constrained by context-specific health system challenges. Addressing these barriers through tailored strategies is essential for achieving effective, scalable, and sustainable integration.

## Abbreviations

CFIR: Consolidated Framework for Implementation Research
FBF: Fortified Blended Food
HEP: Health Extension Program
HEW: Health Extension Worker
IMAM: Integrated Management of Acute Malnutrition
MAM: Moderate Acute Malnutrition
MHNT: Mobile Health and Nutrition Team
MoH: Ministry of Health
MUAC: Mid Upper Arm Circumference
NDRMC: National Disaster Risk Management Commission
PBWs: Pregnant and Breastfeeding Women
PHCU: Primary Health Care Unit
RUSF: Ready to Use Supplementary Food
SAM: Severe Acute Malnutrition
SDG: Sustainable Development Goals
SFFs: Specially Formulated Foods
TAC: Technical Advisory Committee
TSFP: Targeted Supplementary Feeding Program
UNICEF: United Nations Children’s Fund
WDA: Women Development Army
WFP: World Food Program
WHO: World Health Organization
WHZ: Weight for Height Z-score
WoHO: Woreda Health Office
