# Supplementary material for: Understanding barriers and facilitators of integrating management of Moderate Acute Malnutrition (MAM) into the Ethiopian primary health care system using Consolidated Framework for Implementation Research (CFIR)
Source: PLOS Glob Public Health. 2026 Aug 14;6(8):e0007127. doi: 10.1371/journal.pgph.0007127 (PMC13475872; doi:10.1371/journal.pgph.0007127)
Supplement: Supplementary file 1 [file pgph.0007127.s001.docx]

**Reviewer Comments: PGPH-D-26-01051: Understanding barriers and facilitator of integrating management of moderate acute malnutrition (MAM) into Ethiopian health care system using consolidated framework for implementation research (CFIR)**

Thank you for the opportunity to review this manuscript. Overall, I find your manuscript to be well-written, and your study to have much practical relevance to those working to implement complex health interventions within low- and middle-income country contexts. Many of my suggested revisions relate to the presentation of information within your methods and requirement for some additional details. Your discussion also requires a few additional details, as indicated below.

**Minor notes:**

- Please carefully review/revise your submission for typos/language issues. For example, the title requires edits: “Understanding barriers and facilitators of integrating management of moderate acute malnutrition (MAM) into the Ethiopian health care system using the Consolidated Framework for Implementation Research (CFIR)
- A careful edit across the manuscript is required for quite a few typos/minor language issues, particularly in the first few sections
- Should the manuscript be accepted for publication, fairly substantive edits will be required to the reference list, I believe (i.e., missing citation information; inconsistent formatting)

**Abstract:**

- Under ‘Methods’: “CFIR guided the development of the tools, as well as...”: Please specify what tools you are referring to here (i.e., “data collection tools”). I think you’re also missing a word at line 73: “Ratings were used to understand how strongly constructs...”

**Introduction:**

- Overall, this is a well-structured introduction that situates your study within existing relevant literature; provides necessary context on MAM, how it has been managed previously within Ethiopia and the change that has been made with regard to its management; and effectively provides the rationale for your study.
- There are a lot of abbreviations being used which affects readability. Please remove abbreviations for terms that are only being used once and not repeatedly across the manuscript (i.e., WLZ/WHZ; NDRMC; FBFs; RUSFs)

**Methods:**

- Study setting: Can you add a brief phrase to indicate why the MoH, WFP and UNICEF selected the specific implementing districts (i.e., diversity with respect to geography, health system characteristics, etc.?)
  - Lines 178-182: Rather than listing the study districts and their distance from Addis Ababa, I would suggest that a map would help the reader better visualize these areas. If distance from Addis Ababa is useful context for understanding your implementation findings, you could add these specific distances to the map.
- Participants and recruitment: Line 197: Please write the data collection dates out in full for readability, rather than as 15/02/2023 to 05/04/2023.
- Table 1: Given the number of identifiers provided, including the specific role and number of years of service within each specific organization, I would suggest that this information could indirectly identify your KII participants. If not, I would still suggest that this level of detail is not necessary. Instead, synthesize your sections further, and remove the years of service as a column. For example, your rows could include “National level organizational representatives: n=9”; “Regional/subnational administrators: n=16”, etc. Then in the main text, you could still offer a few examples of the organizations represented within your sample, the types of roles individuals held, a range/average number of years of service, etc. but you would not be linking these attributes to specific, individual participants.
  - Reading further, I am noticing you have this information that characterizes/positions your KIIs within the ‘Recruitment of participants section’. I would then suggest either removing Table 1 and adding n values within the main text, or significantly synthesizing the table, as I’ve described above. Currently, the table and text are redundant.
- Data collection: Indicate what virtual platforms were used for data collection.
  - How many different trained research assistants conducted interviews?
  - Please add information about interview duration, i.e., the range of duration across interviews and the average interview duration.
- Data management and analysis: Revise this to “Data analysis” and integrate the first sentence (lines 241-243) to the end of the preceding section where transcription is already being discussed.
  - I don’t think ‘abductive analysis’ is what you are referring to here. You describe your analytic approach in sufficient detail, but to me, your description maps to a “hybrid deductive-inductive analysis” (see Fereday and Muir-Cochrane (2006) reference: <https://doi.org/10.1177/160940690600500107>), beginning with the deductive approach and followed by an inductive analysis using NVivo.
  - If, however, an ‘abductive analysis’ approach is actually more suited to what you did, then you need to clarify what this approach is and why it was chosen as an appropriate qualitative analysis methodology.
  - Table 2: Are these criteria directly from Damschroder and Lowery’s approach, or have you adapted their ratings/criteria in any way for use within your study (or created your own criteria)? This is currently unclear and should be explicitly indicated.
  - Operationalization of CFIR domains: For improved readability/flow, please move this information into a table or box, rather than in the main text.
  - Trustworthiness: While I appreciate your thoughtfulness in presenting the various validity procedures you utilized to enhance rigour/trustworthiness, I would suggest that these individual paragraphs should be collapsed into a single, more synthetic paragraph. You also do not need to explicitly state the meaning of these various dimensions of trustworthiness, as readers familiar with qualitative approaches already understand what these concepts mean. Focus on stating how you applied these specific elements within your analysis procedures.
    - Can also cite Creswell, J. W., & Miller, D. L. (2000). Determining Validity in Qualitative Inquiry. *Theory Into Practice*, *39*(3), 124–130. <https://doi.org/10.1207/s15430421tip3903_2>

**Findings:**

- Unless I missed it, I don’t think TSFP is defined at first use within your manuscript. Please write it out in full at first use (line 339).
- Overall, your findings are well-written. They are well-structured, thorough, yet concise.

**Discussion:** You elaborate on your findings and situate them within relevant literature effectively.

- Your discussion is quite long, and I would suggest that it could be ‘tightened up’ quite considerably
- Your discussion hints at the broader contributions of your study and relevance within other contexts; however, it would be helpful to add a brief paragraph that clearly describes the broader take-aways, both pragmatically for other health system/organizational implementers and also for the research community as this is an academic journal. What are future research directions?
- Please reflect on your study limitations.

I hope my review is useful to you. All the best with your continued work.
